# *Elk3* Deficiency Causes Transient Impairment in Post-Natal Retinal Vascular Development and Formation of Tortuous Arteries in Adult Murine Retinae

**DOI:** 10.1371/journal.pone.0107048

**Published:** 2014-09-09

**Authors:** Christine Weinl, Christine Wasylyk, Marina Garcia Garrido, Vithiyanjali Sothilingam, Susanne C. Beck, Heidemarie Riehle, Christine Stritt, Michel J. Roux, Mathias W. Seeliger, Bohdan Wasylyk, Alfred Nordheim

**Affiliations:** 1 Department of Molecular Biology, Interfaculty Institute for Cell Biology, University of Tuebingen, Tuebingen, Germany; 2 Institut de Génétique et de Biologie Moléculaire et Cellulaire, Illkirch, France; 3 Centre National de la Recherche Scientifique, Illkirch, France; 4 Institut National de la Santé et de la Recherche Médicale, Illkirch, France; 5 Université de Strasbourg, Illkirch, France; 6 Division of Ocular Neurodegeneration, Centre for Ophthalmology, Institute for Ophthalmic Research, University of Tübingen, Tübingen, Germany; Medical University Innsbruck, Austria

## Abstract

Serum Response Factor (SRF) fulfills essential roles in post-natal retinal angiogenesis and adult neovascularization. These functions have been attributed to the recruitment by SRF of the cofactors Myocardin-Related Transcription Factors MRTF-A and -B, but not the Ternary Complex Factors (TCFs) Elk1 and Elk4. The role of the third TCF, Elk3, remained unknown. We generated a new *Elk3* knockout mouse line and showed that Elk3 had specific, non-redundant functions in the retinal vasculature. In *Elk3(−/−)* mice, post-natal retinal angiogenesis was transiently delayed until P8, after which it proceeded normally. Interestingly, tortuous arteries developed in *Elk3(−/−)* mice from the age of four weeks, and persisted into late adulthood. Tortuous vessels have been observed in human pathologies, e.g. in ROP and FEVR. These human disorders were linked to altered activities of vascular endothelial growth factor (VEGF) in the affected eyes. However, in *Elk3(−/−)* mice, we did not observe any changes in VEGF or several other potential confounding factors, including mural cell coverage and blood pressure. Instead, concurrent with the post-natal transient delay of radial outgrowth and the formation of adult tortuous arteries, Elk3-dependent effects on the expression of Angiopoietin/Tie-signalling components were observed. Moreover, *in vitro* microvessel sprouting and microtube formation from P10 and adult aortic ring explants were reduced. Collectively, these results indicate that Elk3 has distinct roles in maintaining retinal artery integrity. The *Elk3* knockout mouse is presented as a new animal model to study retinal artery tortuousity in mice and human patients.

## Introduction

Angiogenesis is an important physiological process in which new blood vessels are generated by sprouting of existing ones. Dysregulated angiogenesis is implicated in many human diseases, including cancer and retinopathies [1], [2]. During angiogenesis, endothelial tip cells at the angiogenic front form numerous filopodia and guide vascularization, whereas stalk cells located behind tip cells are involved in proliferation and vessel extension [3]. Angiogenesis can be readily studied using the mouse retina. Many angiogenic events, such as endothelial cell (EC) proliferation, sprouting, recruitment of mural cells and maturation, occur post-natally and can be followed *ex vivo* on two-dimensional retinal flat-mount preparations [4]. At post-natal day 0 (P0), vascularization starts as a ring-shaped vessel around the optic nerve head. By P4, half of the retina is covered by blood vessels, and around P8, the retina is fully vascularized regarding the primary plexus, while sprouts develop to form the additional deep plexus capillary networks [5]. Site-directed mutagenesis of the mouse genome can be used to identify genes involved in murine retinal vessel angiogenesis and physiology.

Murine retinal angiogenesis requires the SRF transcription factor [6], [7], [8]. SRF is ubiquitously expressed and fulfills many essential functions [9]. Constitutive deletion of *Srf* results in embryonic lethality at E7.5 [10]. Tissue specific, conditional ablation of *Srf* reveals specific functions in skeletal muscle cells, cardiomyocytes, forebrain neurons, hepatocytes, keratinocytes, intestinal smooth muscle cells and endothelial cells (see below) (for review [9]). Tissue and target gene specificity results from differential recruitment of cofactors to SRF [11], [12], [13]. Recruitment of Ternary Complex Factor (TCF) family members (Elk1, Elk3 or Elk4) [13], [14] can lead to the induction of immediate early genes (IEGs) involved in cell cycle entry, whereas recruitment of Myocardin Related Transcription Factors (MRTF-A and MRTF-B) [15], [16], [17] induces the transcription of genes involved in adhesion and motility [11], [18]. Interestingly, these SRF cofactors have different effects on angiogenesis. Murine endothelial *Srf* depletion results in characteristic phenotypes in early post-natal and adult retinae [7], [8], which are mimicked by deletion of *Mrtf-a and Mrtf-b*, but not by single or double deletion of the *Elk1* and *Elk4* genes [8]. The murine phenotypes upon post-natal endothelial depletion of SRF or MRTF-A/-B reflect some pathological features exhibited by human patients suffering from FEVR (familial exudative vitreoretinopathies) and AMD (adult macular degeneration) [8]. Potential contributions to retinal angiogenesis of the third TCF, Elk3, have not yet been reported. Therefore, we have developed a new mouse model for constitutive Elk3 depletion. We show that *Elk3* deletion leads to a distinct retinal phenotype, manifested in transiently delayed post-natal primary plexus formation and lasting tortuous arteries in adult retinae. This does not impair visual function. At the molecular level, *Elk3(−/−)* mice display altered retinal expression of immediate-early genes and angiogenic Tie receptor genes. *Elk3(−/−)* phenotypic features partly resemble human ophthalmologic diseases with tortuous vessels, i.e. retinopathy of prematurity (ROP) [19] and familial exudative vitreoretinopathy (FEVR) [20]. Thus, *Elk3(−/−)* mice promise to be a useful animal model to reveal insufficiently understood pathogenic mechanisms that lead to human retinal vessel tortuousity.

## Materials and Methods

### Generation of *Elk3* constitutive knockout (KO) mice

A previously used mouse model of Elk3 deficiency expresses a truncated form of the protein (Net δ) [21]. To avoid this confounding factor, a new constitutive Elk3 knockout mouse line was established in a cooperation between the IGBMC and the MCI/ICS (Mouse Clinical Institute (Institut Clinique de la Souris), Illkirch, France; http://www.ics-mci.fr). The targeting vector was constructed as follows. The 5′ (4.3 kb), 3′ (3 kb) and interloxP (3 kb) fragments were PCR amplified on 129sv genomic DNA and sequentially subcloned in an ICS proprietary vector containing the LoxP sites and a Neo cassette flanked by FRT sites (Figure S1). The linearized construct was electroporated in 129S2/SvPas mouse embryonic stem (ES) cells. After selection, targeted clones were identified by PCR using external primers and further confirmed by Southern blotting with 5′ external probe. Two positive ES clones were injected into C57BL/6J blastocysts, and derived male chimeras gave germline transmission. The excision of the neomycin-resistance cassette was performed *in vivo* by breeding the chimeras with a Flp deleter line (C57BL/6J genetic background). The Flp transgene was segregated by breeding the first germ line mice with a wild type C57BL/6J animal. Constitutive KO mice were generated by breeding floxed-allele heterozygotes with a Cre deleter line followed by segregation in a further breeding step.

### Experimental animals

To generate *Elk1/Elk4* double knockout (*dKO*) animals, termed *Elk1/Elk4^dKO^*, single *Elk1(−/0)* [22] and single *Elk4(−/−)* [23] founder mice were used. These mouse strains were crossed in matings of *Elk1(−/−)::Elk4(+/−)* females with *Elk1(−/0)::Elk4(+/−)* males [24].

### Antibody staining of retinal flat-mounts *in vitro*

Generation of IsolectinB4 stained retinal flat-mounts was performed as described previously [8]. Briefly, eyes were isolated and fixed in 4% PFA for two hours at RT. After 2×5 minutes in PBS, retinae were dissected and incubated in blocking buffer (1% BSA, 0.3% Triton-X, PBS) for 2 h at RT. Incubation with primary antibodies was performed at 4°C in blocking buffer overnight. After washing 3×20 minutes with PBS, retinae were incubated with secondary antibodies in blocking buffer for two hours at RT. After washing 3×20 minutes in PBS, retinae were flat-mounted on coverslides and embedded in Mowiol for fluorescence microscopy.

Primary antibodies: CollagenIV 1∶40 (AbD Serotec), Ki67 (SP6 undiluted) (DCS). Secondary antibodies: anti rabbit Alexa 546 1∶200 (Molecular Probes), SMA-Cy3 conjugate 1∶200 (Sigma Aldrich). Retinal vessels were stained with Isolectin B4 (ILB4) from Griffonia simplicifolia 1∶25 (Sigma), detected by Streptavidin-Alexa488 1∶100 (Molecular Probes).

### RNA isolation, cDNA synthesis and qRT-PCR analysis of brain and retinal tissue

Brains and retinae of post-natal pups or adult animals were dissected and frozen in liquid nitrogen for further use. All tissues were lysed for RNA isolation according to the manufacturer's protocol (Qiagen, RNeasy). cDNA synthesis was performed using random hexamers. qRT-PCR analysis was performed using specific primers (Sigma) and SYBR green technology in an ABI Prism 7000 cycler. Primer mix for one sample included 10 µM forward primer (0.3 µl), 10 µM reverse primer (0.3 µl) and 2.4 µl water. cDNA mix for one sample included 5 µl SYBR green and 2 µl cDNA. For each qRT-PCR reaction, 7 µl of the cDNA mix and 3 µl of the primer mix were combined in the 96-well plates. Primer sequences are listed in Table S1. For description of general methods, see [25]. Amplification protocol: segment 1: 50°C, 2 min., segment 2: 95°C, 10 min., segment 3: 95°C, 15 sec, 60°C, 1 min., 40 cycles.

### Western Blot

Retinal tissue was lysed in Iyer-buffer (0.5 M Hepes pH 7.5; 1 M MgCl_2_; 0.5 M EDTA; 5 M NaCl; in water) [26]. Protein content of cell lysates was determined by Bradford reagent. Separation of bands was performed by SDS-PAGE (12% gel for SMA, 15% for P-Cofilin). Electrotransfer was done at 4°C for 2 h at 100 V and 400 mA. To detect specific bands, membranes were blocked in 5% bovine serum albumin BSA for one hour at RT. Incubation in primary antibodies was performed overnight at 4°C. After three washes with TST (Tris Saline Tween), membranes were incubated in secondary antibodies for one hour at RT. Primary antibodies: GAPDH 1∶20000 (Hytest Ltd.), P-Cofilin 1∶500 (Cell Signalling), SMA 1∶1000 (Sigma Aldrich). Secondary antibodies (1∶10000 dilution, GE Healthcare): anti-mouse IgG HRP-conjugated, anti-rabbit IgG HRP-conjugated.

### Scanning laser ophthalmoscopy (SLO)

Scanning-laser ophthalmoscopy (SLO) was performed as described previously [27]. Briefly, mice were anaesthetized by subcutaneous injection of ketamine (66.7 mg/kg) and xylazine (11.7 mg/kg). After anaesthesia, pupils were dilated with tropicamide eye drops (Mydriaticum Stulln, Pharma Stulln, Stulln, Germany). SLO imaging was performed using a Heidelberg Retina Angiograph (HRA I) equipped with an argon laser featuring two wavelengths (488 nm and 514 nm) in the short wavelength range and two infrared diode lasers (795 nm and 830 nm) in the long wavelength range. The laser wavelength used for fundus visualization was 514 nm (RF, red-free channel). The 488 nm wavelength was used for fundus autofluorescence (AF) analysis. Additionally, the 488 nm and 795 nm lasers were used for fluorescein angiography (FLA) and indocyanine green angiography (ICGA), respectively. FLA and ICGA were performed using subcutaneous injection of 75 mg/kg body weight fluorescein-Na (University pharmacy, University of Tübingen, Germany), or 50 mg/kg body weight ICG (ICG-Pulsion, Pulsion Medical Systems AG, Munich, Germany), respectively.

### Electroretinography (ERG)

Electroretinograms were recorded as described previously [28]. After overnight dark-adaptation, single-flash ERG responses were obtained under scotopic (dark-adapted; no background illumination) and photopic (light-adapted with a background illumination of 30 cd/m^2^, starting 10 min before recording) conditions. Single white-flash stimuli ranged from −4 to 1.5 log cd s/m2 under scotopic and from −2 to 1.5 log cd s/m^2^ under photopic conditions. Ten responses were averaged with inter-stimulus intervals of 5 s (for −4 to −0.5 log cd s/m^2^) or 17 s (for 0 to 1.5 log cd s/m^2^).

### Hematoxylin/Eosin (H&E) staining of paraffin sections of whole eyes

Eyes were fixed overnight at 4°C in Davidson's fixative (6% formaldehyde, 32% ethanol, 11% acetic acid, 5% sucrose in PBS). Histological examination of the eyes was performed on 4 µm sections of paraffin embedded eyes mounted on Superfrost Plus slides (Langenbrinck, Emmendingen, Germany). Sections were stained with Hematoxylin/Eosin followed by dehydration and mounting in Entellan.

### Aortic ring assay

Aortae from P10 or adult *Elk3(+/+)* control and *Elk3(−/−)* knockout animals were excised, cut into 1 mm rings and embedded in Matrigel (BD Biosciences) on cleaned coverslips (12 mm diameter) in 4-well plates. Matrigel was polymerized for 10 minutes at RT followed by 30 minutes at 37°C. Embedded rings were covered with 700 µl of HUVEC EGM growth medium with all required supplements added according to the manufacturer's protocol (Lonza). Fresh medium was supplied every other day. Aortic rings from P10 and adult mice were cultivated for two and three weeks, respectively, and microvessel sprouting of each aortic ring was quantified every day using the following score: 0 =  no microvessel sprouting, 0.25 =  isolated sprouting, 0.5 = 20–50% sprouting, 0.75 = 50–75% sprouting, 1 = 75–100% sprouting, 1.25 = 100% sprouting. A tip cell index for microvessel sprouting was calculated for each day and plotted to reveal the kinetics of microvessel sprouting for each genotype. To stain actin filaments of embedded aortic rings, rings were fixed with 4% PFA, washed 3× with PBS, permeabilized with 10% Triton, blocked with 2% BSA, and incubated with Phalloidin-Texas Red. Rings were washed 3× with PBS and photographed on coverslips, which were removed from the 4-well plates.

### Microscopic analysis

**Figure 1:**
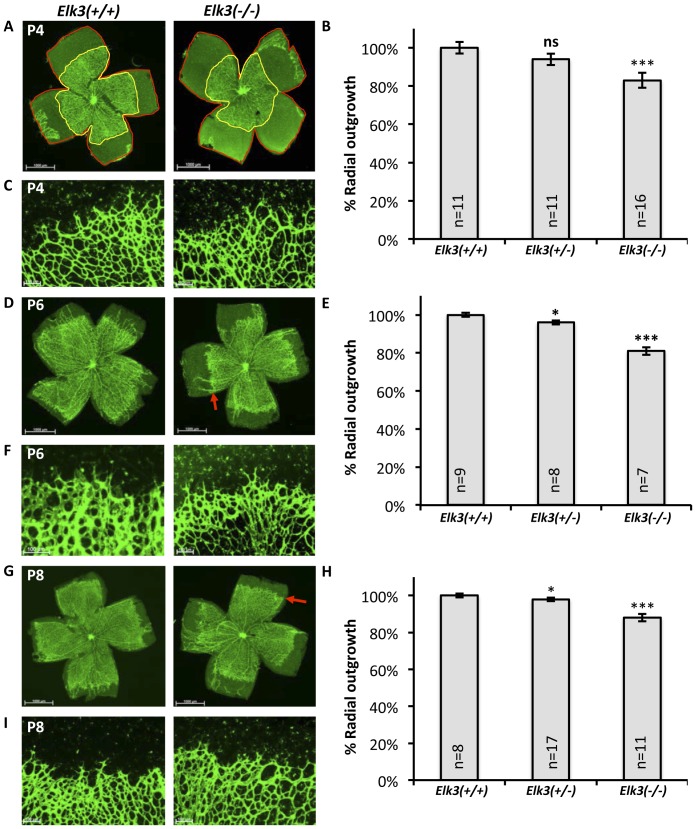
Deletion of *Elk3* leads to delayed retinal angiogenesis during early post-natal stages in *Elk3(−/−)* knockout mice. (A) IsolectinB4 staining of flat-mounts of retinae from P4 *Elk3(+/+)* control and *Elk3(−/−)* knockout mice. The red lines outline the whole retinal area, the yellow lines the areas covered by blood vessels. (B) Quantitation of retinal area covered by blood vessels (% vascularization) at P4. (C) Higher magnification of the angiogenic front in P4 control and *Elk3(−/−)* knockout retinae. (D) IsolectinB4 staining of retinal flat-mounts of a P6 *Elk3(+/+)* control and *Elk3(−/−)* knockout mouse. The red arrow points towards delayed angiogenic front in the *Elk3(−/−)* retina. (E) Quantitation of retinal area covered by blood vessels (% vascularization) at P6. (F) Higher magnification of the angiogenic front in P6 control and *Elk3(−/−)* knockout retinae. (G) IsolectinB4 staining of retinal flat-mounts from P8 *Elk3(+/+)* control and *Elk3(−/−)* knockout mice. The red arrow points towards the delayed angiogenic front in the *Elk3(−/−)* retina. (H) Quantitation of retinal area covered by blood vessels (% vascularization) at P8. (I) Higher magnification of the angiogenic front in P8 control and *Elk3* knockout retinae. All quantitation data are normalized to the control  =  100%. The data shown are means +/− s.e.m. Statistical significance using Student's t-test is indicated with * p<0.05 ** p<0.01 *** p<0.001. Scale bar in (A, D, G) 1000 µm, in (C, F, I) 100 µm.

For fluorescent staining analysis, a Zeiss Axiovert 200 M microscope equipped with an AxioCam MRm camera and an ApoTome (Zeiss) was used. Retinal overviews (original magnification 5×) are presented as composite images of individual, successively overlapping (5%) images, generated by computer-controlled x-y settings and processed using MosaiX Software. H&E-stained sections were visualized using Zeiss Axioplan 2 with AxioCamHRc camera. Higher magnifications were obtained with 10× and 20× objectives. % radial outgrowth, a quantitative measure for retinal vascularization, was calculated by dividing the retinal area covered by blood vessels (yellow line in Figure 1A) by the total retinal area (red line in Figure 1A). The percentage of abnormally shaped arteries was calculated by counting both abnormally shaped arteries and the total number of arteries, using SLO imaged retinae and retinal flat-mounts. Mean blood vessel width was calculated as follows: blood vessels were identified by ILB4 staining followed by measuring blood vessel areas and blood vessel lengths. Blood vessel widths (W) were calculated dividing vessel length (L) by area (A), i.e. W = L/A. Tortuousity of adult vessels was quantified as shown in Figure S2. A tortuousity factor was calculated by normalizing actual vessel length (red line) over idealized vessel length (yellow line), as measured between common equidistant endpoints. For quantitation of proliferation in arteries and veins, retinal flat-mounts of *Elk3(+/+)* WT and *Elk3(−/−)* KO P6 animals were co-stained with ILB4 and Ki67 and photographed at 20× magnification with focus on single arteries and veins. Using these images, Ki67 positive endothelial cells were counted and normalized to 100 µm vessel length. Obtained values are expressed as % of WT values (Table S2).

### Statistical analysis

For all quantitative analyses, data are presented as means +/− s.e.m. For comparison of different experiments, values are normalized to the control  = 100%. To test significance, Student's t-tests were used, p levels<0.05 were considered significant. Significance is indicated by * p<0.05, ** p<0.01, *** p<0.001 and ns (not significant).

### Study approval

All animal experiments were approved by the Regierungspräsidium Tübingen (Tübingen, Germany) permit for SLO angiography of wt and *Elk3(−/−)* mice (IM 1/13, approved 20^th^ February 2013, valid until 28^th^ February 2016), permit for SLO imaging of wt and *Elk1/Elk4^dko^* mice (IZ 1/13, approved 15^th^ August 2013, valid until 31^st^ August 2016), and permit for ERG measurements of wt and *Elk3(−/−)* mice (IM 3/13, approved 06^th^ December 2013, valid until 15^th^ December 2016).

## Results

### Generation of *Elk3* deficient constitutive knockout mice

Elk3 deficient mice were generated by homologous recombination and subsequent Cre-mediated deletion, as described in Materials and Methods and Figure S1. The *Elk3* gene was deleted around the major transcription start and exon 1, from −1720 to + 650 relative to the major transcriptional start site. The deletion decreased Elk3 expression at the RNA level, by at least 99.5% in the retina (see below) and other adult tissues and embryos (E8.5, 10.5, 12.5 and 14.5; data not shown), and at the protein level in E12.5 mouse embryo fibroblasts (data not shown).

### General description of phenotypic features of *Elk3* knockout mice

The *Elk3* homozygous knockout mice displayed impaired viability with variable penetrance (usually around 50% of the expected ratio at P10 for heterozygous crosses in this study). The reasons for this partial lethality were not systematically examined, but chylothorax was observed, as in the previous study with the Net δ mouse [21] (and data not shown). Surviving 8 week old mice were globally phenotyped by the EUMODIC Pipelines 1 & 2 (see EMPRESS). The most significant (p<0.001) annotation in both Pipelines (PipelinesElk3) was vessel pattern detected by indirect ophthalmoscopy (see below). There was no change in systolic arterial pressure and pulse rate that could be detected by the non-invasive blood pressure tests (Figure S3). These “high throughput” observations suggested that the “retinal vascular structure” defect was a prime candidate for further investigation, especially given the role in this tissue of the Elk3 interacting partner SRF [7], [8].

### *Elk3* deficiency results in reduced vascularization during early post-natal retinal angiogenesis

Guided by the observations obtained after endothelial-specific *Srf* deletion, we analysed the effect of Elk3 depletion on post-natal retinal angiogenesis since, during this period, SRF depletion resulted in reduced vascularization of the retina, formation of distal microaneurysms at the angiogenic front, and absence of deep plexi [8]. Observation of IsolectinB4 stained retinal flat-mounts at post-natal stages P4, 6, and 8 showed that retinal primary plexus vascularization was reduced in *Elk3(−/−)* animals (Figures 1A, D and G; quantifications in Figures 1B, E and H, red arrows point towards delayed angiogenic fronts in P6 and P8 *Elk3(−/−)* retinae). However, higher magnification views of angiogenic fronts did not reveal any abnormalities of tip cell morphology and number and length of tip cell filopodia in *Elk3(−/−)* knockout retinae (Figure 1C, F, I), suggesting that the phenotype is different from *Srf ^iECKO^* animals, as well as from *Mrtf-a^(−/−)^Mrtf-b^iECKO^* animals [8].

### Reduced angiogenesis in *Elk3* knockout retinae is transient and is overcome at post-natal day 10

**Figure 2:**
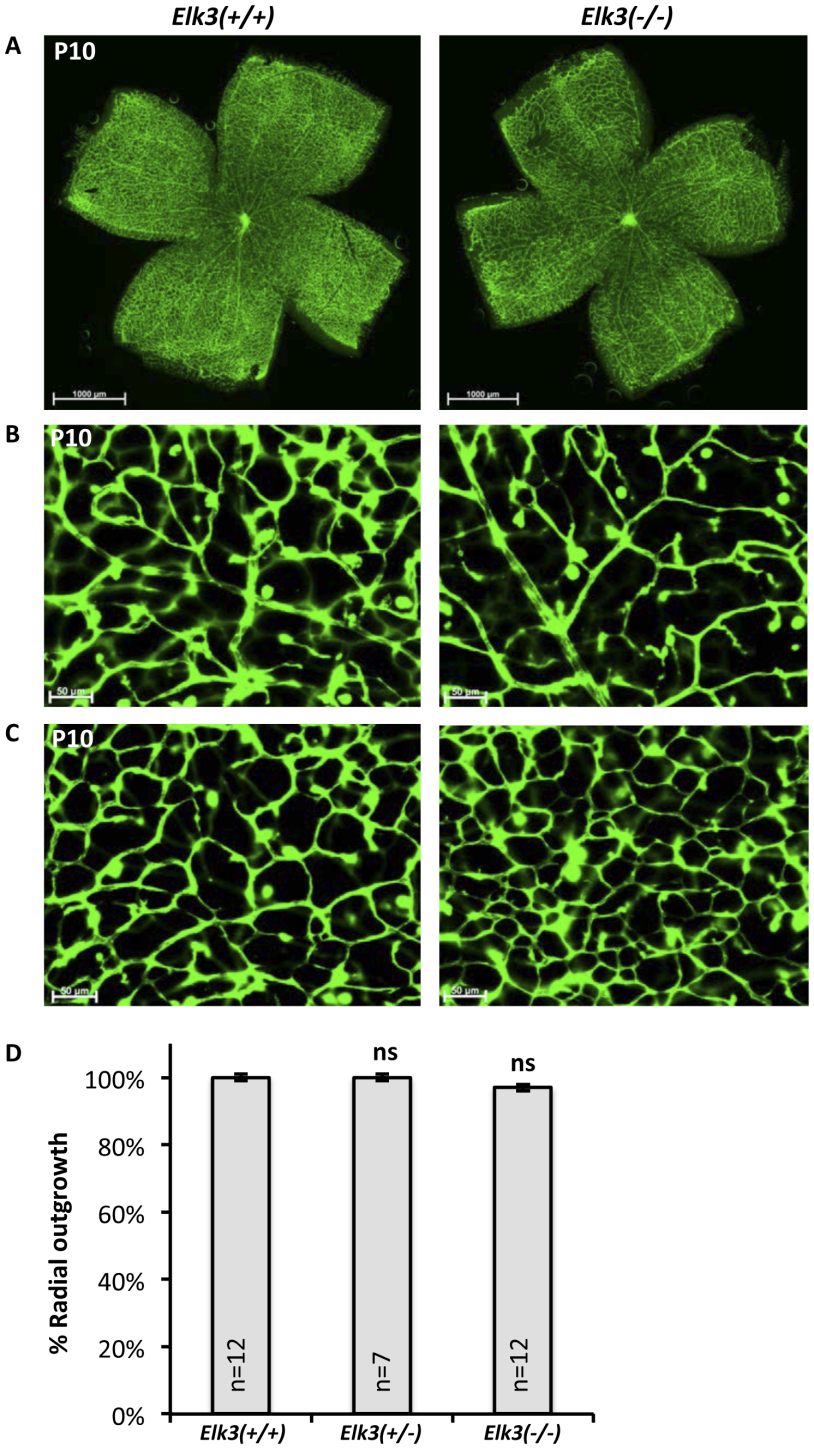
At P10, retinal vascular plexi appear normal in *Elk(−/−)* knockout mice. (A) Representative images of IsolectinB4 stained retinal flat-mounts from P10 control and *Elk3(−/−)* knockout mice. (B) Focal plane set on the primary plexus in control and *Elk3(−/−)* knockout retinae. (C) Focal plane set on the deep plexus in control and *Elk3(−/−)* knockout retinae. (D) Quantitation of retinal area covered by blood vessels (% vascularization) at P10. Data are normalized to the control  = 100%. The data shown are means +/− s.e.m, ns  =  not significant. Scale bar in (A) 1000 µm, in (B, C) 50 µm.

The delay in radial outgrowth of the primary plexus was mild, suggesting that it could be overcome with time. Indeed, the outgrowth at post-natal day 10, observed on IsolectinB4 stained retinal flat-mounts, was indistinguishable in *Elk3(+/+)* control and *Elk3(−/−)* knockout retinae (Figure 2A and quantitation of radial outgrowth in Figure 2D). There were no avascular zones in the primary retinal periphery, and deep plexi formed normally in both control and *Elk3(−/−)* knockout retinae (Figure 2B and 2C). These findings indicated that impaired radial outgrowth at P4/P6/P8 was transient and was overcome by P10 in *Elk3(−/−)* knockout animals.

### Scanning laser ophthalmoscopy reveals tortuous arteries in *Elk3* deficient, but not in *Elk1/Elk4* deficient, adult retinae

**Figure 3:**
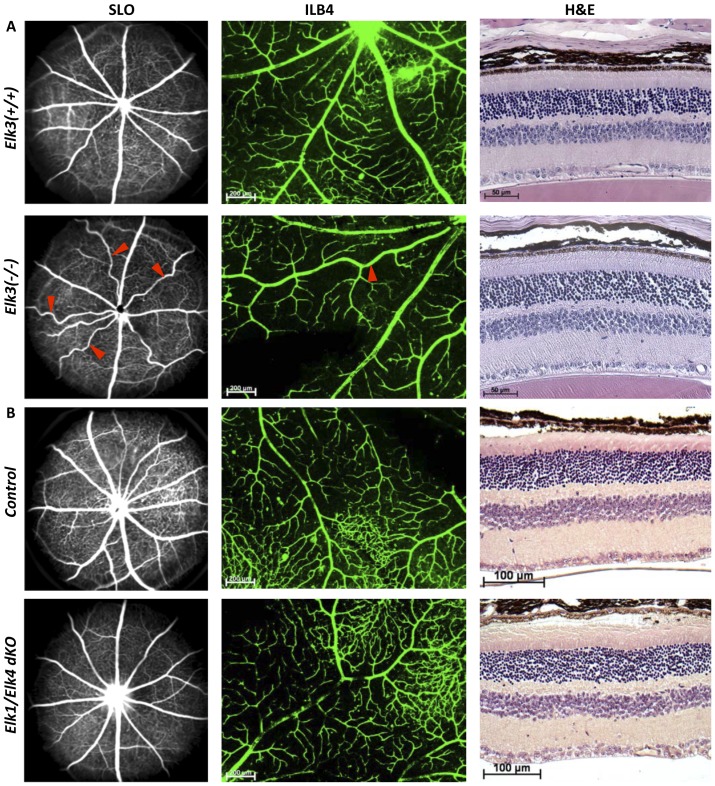
Scanning laser ophthalmoscopy reveals abnormally shaped tortuous arteries in adult *Elk3(−/−)*, but not *Elk1/Elk4* ^dKO^ eyes. (A) Scanning laser ophthalmoscopy (SLO) of *Elk3(+/+)* control (upper panel) and *Elk3(−/−)* knockout (lower panel) retinae by indocyanin green angiography (left) and a higher magnification of arteries in IsolectinB4 stained retinal flat-mounts of the same retina (middle; tortuous arteries are highlighted by red arrowheads). H&E staining of paraffin embedded whole eyes (right). (B) Scanning laser ophthalmoscopy (SLO) of *Elk1/Elk4* control (upper panel) and *Elk1/Elk4^dKO^* (lower panel) retinae by indocyanin green angiography (left) and a higher magnification of arteries in IsolectinB4 stained retinal flat-mounts of the same retina (middle). H&E staining of paraffin embedded whole eyes (right). Scale bar in (middle) 200 µm, in (A right) 50 µm, in (B right) 100 µm.

Adult *Srf ^iECKO^* animals have neovascular lesions that connect to the retinal pigment epithelium, which can be detected by *in vivo* SLO imaging of adult eyes and confirmed by subsequent H&E staining of paraffin sections [8]. We therefore studied the effects of Elk3 deficiency using the same techniques. With SLO imaging, we found that arteries of *Elk3(−/−)* animals exhibited an abnormal tortuous morphology, which was not observed in control animals (Figure 3A left; tortuous arteries in the knockout retina are highlighted by red arrowheads). These findings were confirmed on IsolectinB4 stained retinal flat-mounts prepared after completion of *in vivo* imaging (Figure 3A middle). The penetrance of the tortuous arterial phenotype was 100%, i.e. all *Elk3(−/−)* knockout animals showed tortuousity of arteries in their retinae. We studied the effect of age on the proportion of abnormally shaped arteries. Two-week old animals did not have any detectable abnormalities (data not shown). Arterial tortuousity was detected as early as 4 weeks after birth and increased with age (Table 1). Heterozygotes also exhibited this phenotype, which was however less pronounced (Table 1). Tortuousity was quantified by measuring the lengths of arteries and calculation of a tortuousity factor (see Material and Methods, and Figure S2). *Elk3(−/−)* knockout arteries were significantly longer than control *Elk3(+/+)* arteries (Table 2). In contrast, there were no changes in vessel widths (see Material and Methods, and Table 2). Retinal layering and layer thickness, as determined by H&E staining on paraffin sections of whole eyes, were similarly unaffected. In addition, neovascularization was not observed in *Elk3(−/−)* knockout retinae (Figure 3A right), in contrast to adult *Srf ^iECKO^* animals [8].

**Table 1:** Incidence (%) of tortuous arteries.

Age/months	*Elk3(+/+)* control	*Elk3(+/−)* heterozygotes	*Elk3(−/−)* KO
**1**	0% (n = 7)	12% (±5%) (n = 8) *	62% (±11%) (n = 5) ***
**2**	0% (n = 6)	11% (±5%) (n = 8) **	91% (±7%) (n = 5) ***
**≥4**	3% (±3%) (n = 5)	44% (±9%) (n = 10) ***	85% (±8%) (n = 5) ***
	***Elk1/Elk4*** ** control**	***Elk1/Elk4*** ** heterozygotes**	***Elk1/Elk4*** **^dKO^**
**≥4**	0% (n = 4)	na	0% (n = 5) ns

(* p<0.05 ** p<0.01 *** p<0.001, na = not analysed, ns = not significant, n = number of retinae analysed).

**Table 2:** Extent of tortuousity and width of adult arteries.

	*Elk3(+/+)* control	*Elk3(−/−)* KO	*Elk1/Elk4* control	*Elk1/Elk4* ^dKO^
**Tortuousity factor**	1.0 (±0.003) (n = 9)	1.06 (±0.008) (n = 9) ***	1.0 (±0.001) (n = 6)	0.999 (±0.002) (n = 5) ns
**Width of adult arteries**	20.5 µm (±1.0) (n = 9)	20.6 µm (±1.0) (n = 9) ns	19.7 µm (±1.0) (n = 6)	20.3 µm (±1.06) (n = 5) ns

(*** p<0.001, ns = not significant, n = number of retinae analysed).

To determine if deficiency of the two other TCFs, Elk1 and Elk4, had similar effects as Elk3 deficiency, we analysed control and *Elk1/Elk4^dKO^* adult animals by the same techniques. No abnormalities were detected in *Elk1/Elk4^dKO^* retinae by *in vivo* imaging and on retinal flat-mounts stained with IsolectinB4 (Figure 3B left and Figure 3B middle). There were no changes in arterial shapes, lengths and widths (Table 2), nor in retinal layering and layer thickness (as judged by H&E staining on paraffin sections of whole eyes, Figure 3B right). Moreover, neovascularizations were not detected in *Elk1/Elk4^dKO^* retinae, in contrast to adult *Srf ^iECKO^* animals. Taken together, these data indicate that the *Elk3(−/−)* phenotype of tortuous retinal arteries is distinct from Elk1 and Elk4 deficiencies in the *Elk1/Elk4^dKO^* mice, and SRF deficiency in *Srf ^iECKO^* animals. This reveals a specific role of Elk3 in retinal vessel formation, which is non-redundant to the TCF paralogues Elk1 and Elk4. Analysis of visual function by ERG in adult *Elk3(+/+)* control and *Elk3(−/−)* knockout mice did not detect any impairment, suggesting that *Elk3(−/−)* mice can see normally despite their abnormally shaped arteries (Figure S4).

### Molecular and cellular defects in post-natal *Elk3* deficient retinae

**Figure 4:**
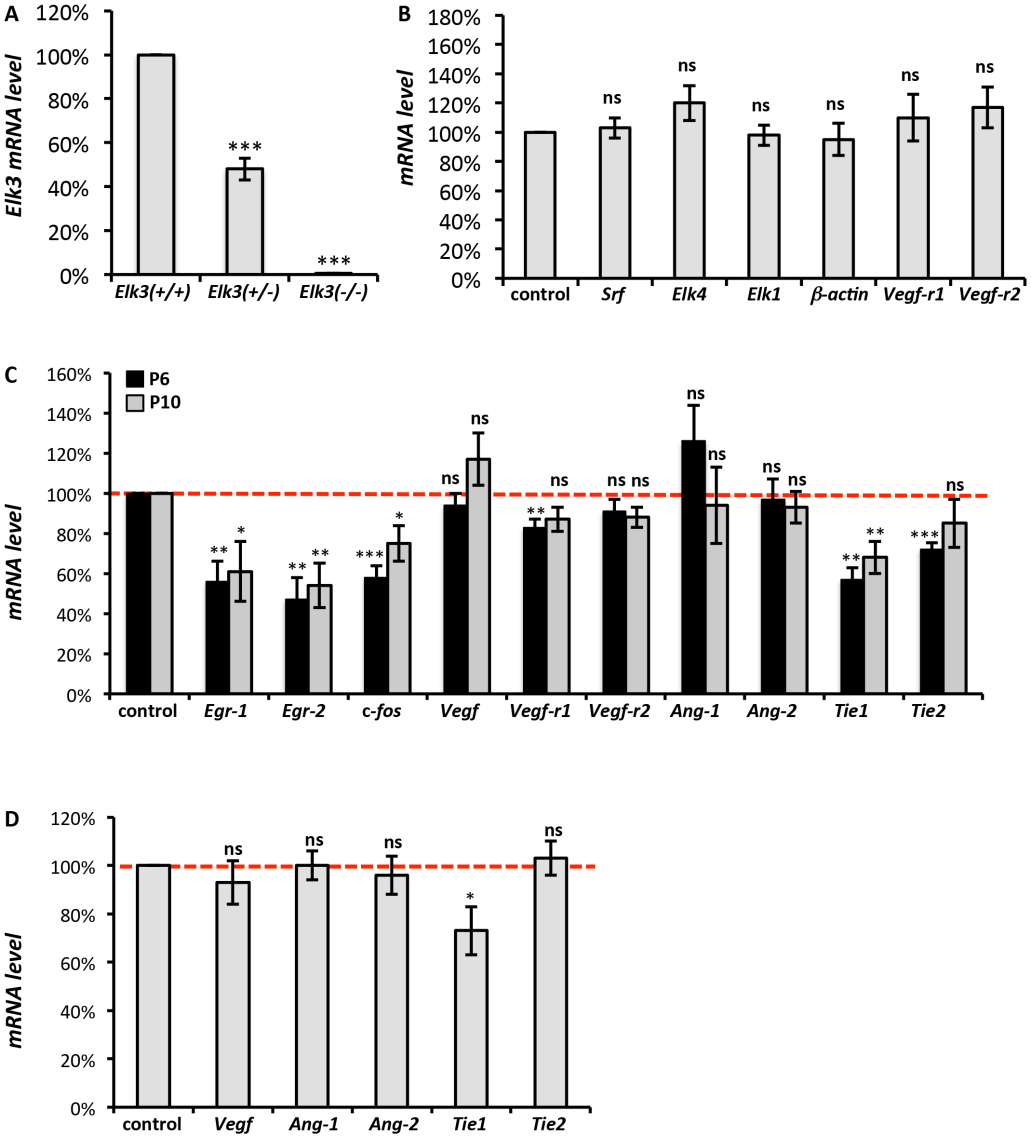
Downregulation of immediate-early genes and the angiogenic Tie1 receptor gene in post-natal *Elk3(−/−)* retinae. (A) Quantitative RT-PCR analysis of whole brain lysates of *Elk3(+/+)* control, *Elk3(+/−)* heterozygote and *Elk3(−/−)* knockout P4-P10 animals for expression of *Elk3* RNA level (n = 9 independent experiments). (B) Semiquantitative RT-PCR analysis of whole brain lysates of *Elk3(+/+)* control and *Elk3(−/−)* knockout P6 animals for *Srf, Elk1, Elk4, β-actin, Vegfr-1, Vegfr-2* expression level (n = 5 independent experiments). (C) Quantitation of RNA levels in P6 and P10 control and *Elk3(−/−)* knockout retinae for *Vegf, Egr-1, Egr-2, c-fos, Vegfr-1, Vegfr-2, Ang-1, Ang-2, Tie1* and *Tie2* (n>5 independent experiments). (D) Quantitation of *Vegf, Ang-1, Ang-2, Tie1* and *Tie2* RNA levels in adult control and *Elk3(−/−)* knockout retinae (n>5 independent experiments).

To study the underlying molecular mechanisms for the observed phenotypes of Elk3 deficient mice, we studied candidate target-gene expression by quantitative RT-PCR. In post-natal brains of *Elk3(−/−)* animals, *Elk3* RNA levels were <0.5% when compared to wild type, and 48% in *Elk3(+/−)* heterozygotes (Figure 4A). There were no changes in *Elk3* knockout brains of the related factors *Srf, Elk1* and *Elk4*, or of the potential target genes *β-actin* and *Vegfr-1* and *Vegfr-2* (Figure 4B).

In P6 retinae from *Elk3* knockouts, *Elk3* levels were greatly decreased (<0.2%, data not shown), the immediate-early genes (IEGs) *Egr-1, Egr-2* and *c-fos* were significantly downregulated, and regarding angiogenic factors, *Vegf* and *Vegf-r2* were unchanged, while *Vegfr-1* was slightly downregulated (Figure 4C). These data suggested that decreased IEG expression and possibly reduced proliferation could account for the delay in vascular outgrowth during the early post-natal period. However, when investigating this possibility, we did not detect any differences in width (measured microscopically at P6 and P8) and proliferative activity (measured by Ki67 staining at P6) of either arteries and veins in *Elk(−/−)* compared to control retinae (Table S2).

In *Elk3(−/−)* retinae of the later time point P10, when the transient delay in retinal vascularization was overcome, RNA expression level of *Vegfr-1* was restored to wild-type levels, whereas *Egr-1, Egr-2* and *c-fos* were still reduced, similar to P6 (Figure 4C).

To uncover potential causes for the observed delay in vascular outgrowth of *Elk3(−/−)* retinae, we studied RNA expression patterns of the second major class of angiogenic signalling partners, the Angiopoietins and their Tie-receptors Tie1 and Tie2. Interestingly, at P6, both Tie1 and Tie2 were significantly downregulated, whereas the Angiopoietins Ang-1 and Ang-2 were unchanged (Figure 4C). In contrast, at the later stage of P10, only Tie1 was significantly downregulated while Tie2 was restored to wildtype levels (Figure 4C).

We next investigated whether the transiently impaired primary plexus formation in *Elk3(−/−)* retinae was possibly due to impaired migration of astrocytes towards the periphery. We found that astrocytic radial outgrowth, at all stages analysed (P4, P6, P8), was unaffected by the knockout of *Elk3*, as judged by glial fibrillary acidic protein (GFAP) staining on retinal flat-mounts (Figure S5).

Reduced retinal vascularization could be a result of enhanced vessel regression. We therefore performed co-staining of ILB4-positive retinal blood vessels and extracellular collagenIV, using P6 retinal flat-mounts of Elk3 wildtype and knockout mice. Thus we checked for so-called ‘empty sleeves’ of regressed, previously existing vessels, as identified by exclusive collagenIV positivity. At the angiogenic front we did not find any vessel regression (Figure S6). Inside the primary plexus, occasional vessel regression (Figure S6, white arrows) was normalized to plexus area and found not to be significantly different between *Elk3* genotypes (Figure S6; for measurements, see Materials and Methods).

SRF deficiency in neurons of the forebrain results in increased Ser3 phosphorylation of the actin-severing protein cofilin [29]. To analyze whether this indicator of imbalance in actin dynamics was changed in *Elk3(−/−)* knockout retinal ECs compared to controls, similar to *Srf ^iECKO^* endothelial cells [8], we performed Western blotting using a P-Cofilin antibody on adult retinal lysates (Figure S7A). No change in P-Cofilin levels were detected after quantitation of five pairs of control and knockout samples (Figure S7B) indicating that actin imbalance is not the reason for disturbed retinal vascular development.

### Molecular and cellular defects in adult *Elk3* deficient retinae

Various mechanisms could account for the observed formation of tortuous arteries in adult *Elk3(−/−)* animals (see Discussion). We tested for VEGF levels in affected retinae, increased blood pressure, altered coverage of retinal blood vessels with smooth muscle cells and procollagen type IVα1, and expression of the tight junctional components claudin1 and claudin5. Further, since knockout mice of the angiogenic factor Ang-2 displayed arteriolar tortuousity [30], we studied retinal expression of the Ang-1/-2 system, including Tie1 and Tie2 co-receptors.

**Figure 5:**
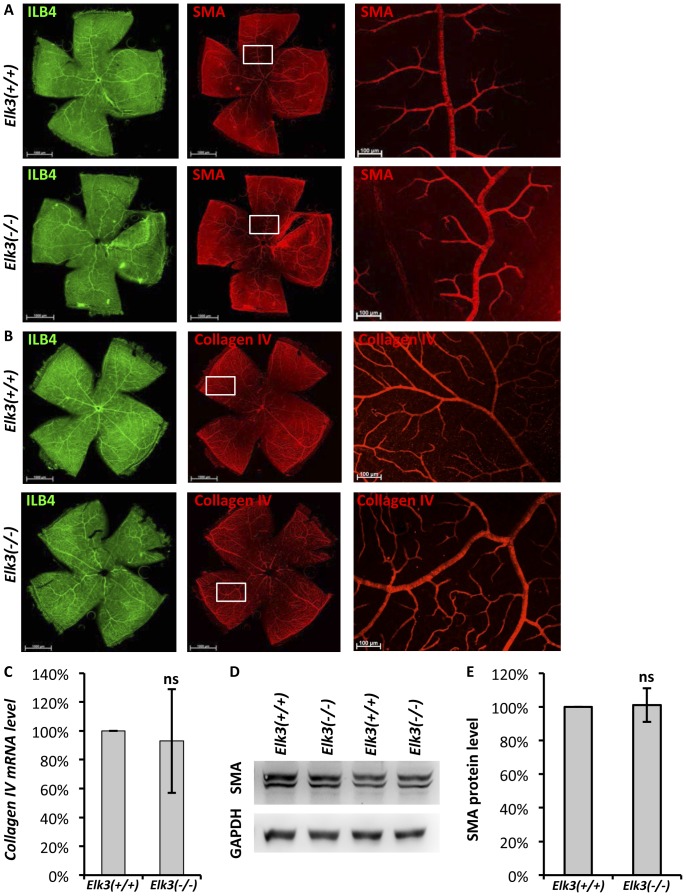
Smooth muscle actin and CollagenIV levels are not altered in *Elk3(−/−)* knockout retinae. (A) IsolectinB4 (ILB4) (left) and smooth muscle actin (SMA) (middle and right) staining of retinal flat-mounts of control (upper panel) and *Elk3(−/−)* knockout animals. (B) IsolectinB4 (left) and collagenIV (middle and right) staining of retinal flat-mounts of control (upper panel) and *Elk3(−/−)* knockout animals. (C) Quantitation of collagenIV RNA levels in control and *Elk3(−/−)* knockout retinae (n = 5 independent experiments). (D) Representative pairs of control and *Elk3(−/−)* knockout retinal lysates tested for smooth muscle actin expression in Western Blot analysis. (E) Quantitation of control and *Elk3(−/−)* knockout retinal levels of SMA (n = 5 independent experiments). Scale bar in (A, B left and middle) 1000 µm, in (A, B right) 100 µm.

We did not detect any significant changes of *Vegf* RNA level in adult *Elk3* knockout retinae (Figure 4D). Interestingly, amongst Angiopoietins and cognate Tie receptors, a significant change was observed for Tie1, as was already found at post-natal ages P6 and P10 (compare Figure 4C and 4D). There were no significant changes in systolic blood pressure, measured by the tail cuff method (Non Invasive blood pressure) (Figure S3). We did not detect any differences in smooth muscle actin (SMA) coverage as judged by antibody staining on retinal flat-mounts (Figure 5A) and analysis of SMA levels by Western blot (Figure 5D and quantitation in Figure 5E). Mutations in the mouse *Col4a1* gene, encoding procollagen type IVα1, resulted in tortuous arteries in the C57BL/6J background [31]. However, we did not detect any significant changes by collagenIV antibody staining of adult retinal flat-mounts (Figure 5B) and qRT-PCR of whole retinal adult tissue (Figure 5C). In *Drosophila*, mutation of the *megatrachea* gene, which plays an essential role in the tracheal system of invertebrates, results in elongated and tortuous tracheal branches in mutant embryos [32] reminiscent of the tortuous vessel phenotype in *Elk3(−/−)* knockout mice. *Mega* encodes a transmembrane protein that is located in septate junctions and is thereby similar in structure and function to claudins, the component of tight junctions in vertebrates. We therefore analysed if claudins were changed in adult *Elk3(−/−)* knockout retinae but did not find any significant changes on RNA levels of claudin1 and claudin5 (data not shown).

### Impairment in *in vitro* microvessel sprouting and microtube formation of aortic ring explants derived from post-natal and adult *Elk3* deficient mice

**Figure 6:**
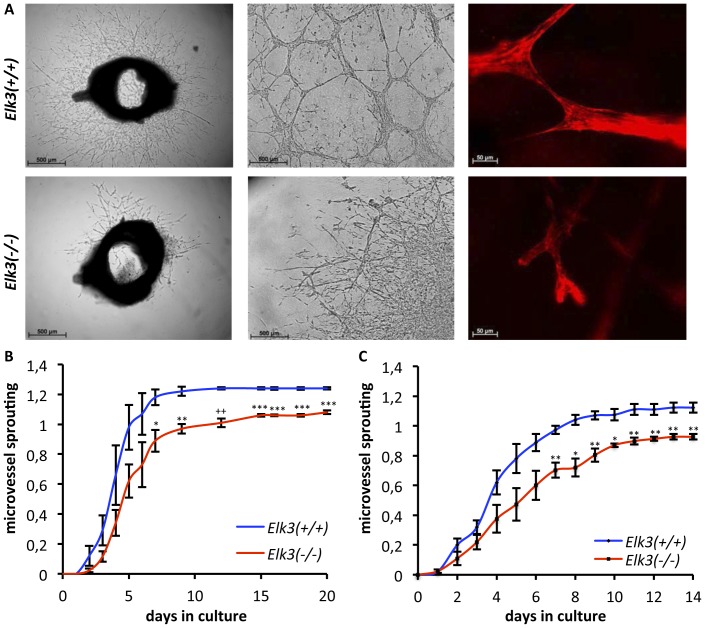
Microvessel sprouting and microtube formation is impaired in P10 and adult *Elk3(−/−)* knockout aortic ring explants. (A left) Aortic rings from *Elk3(+/+)* control animals show robust microvessel sprouting on day 5 of culture. In contrast, aortic rings from *Elk3(−/−)* knockout animals show drastically reduced microvessel sprouting. (A middle) Control *Elk3(+/+)*, but not *Elk3(−/−)* knockout aortic ring endothelial cells form microtubes in Matrigel after three weeks of culture. (A right) Phalloidin staining of aortic ring endothelial cells cultivated in Matrigel shows interconnected ECs and tip cells with filopodia in control explants, whereas *Elk3(−/−)* knockout aortic ring endothelial cells lack connections and instead have degenerated bulbs. (B, C) Kinetics of aortic ring microvessel sprouting for four pairs of adult (B) and four pairs of P10 (C) *Elk3(+/+)* control and *Elk3(−/−)* knockout animals observed during *in vitro* growth (for each time point mean +/*−* s.e.m. presented). Scale bar in (A left and middle) 500 µm, in (A right) 50 µm.

The aortic ring assay is a commonly used assay of angiogenesis. It bridges the gap between *in vivo* and *in vitro* models of angiogenesis, thereby combining advantages of both systems [33]. Endothelial cells first appear at the severed edges of the explants after 2–3 days in culture and subsequently proliferate and migrate thereby resulting in microvessels around the explants. Previous studies using the hypomorphic Net^δ/δ^ mutant mouse line indicated that Elk3 was required for aortic ring sprouting [34]. Using the aortic ring assay, we investigated whether the observed retinal phenotypic abnormalities reflected altered characteristics of endothelial cells in general or represented specific effects of retinal endothelial cells. Aortic rings were embedded in a polymerized extracellular matrix gel supplemented with endothelial growth medium. Microvessel sprouting from *Elk3(−/−)* knockout adult aortic explants was found to be reduced compared to *Elk3(+/+)* control animals (Figure 6A left). A scoring system was used to quantitate and follow the kinetics of microvessel sprouting (see Material and Methods). Sprouting from *Elk3(−/−)* aortic rings was reduced at all time points examined. Sprouting from *Elk3(+/+)* control explants reached a maximum after seven days, whereas *Elk3(−/−)* explants did not reach this level even after 20 days (Figure 6B). Microtube formation after three weeks was robust in *Elk3(+/+)* control explants cultures and greatly reduced in *Elk3(−/−)* cultures (Figure 6A middle). Staining of the cytoskeleton of fixed aortic explants after three weeks *in vitro* was used to study the microtubes. *Elk3(+/+)* control explants had intact interconnections between endothelial tubes and filopodia; whereas *Elk3(−/−)* explants had degenerated endothelial tip cells with retraction bulbs and lacked filopodia (Figure 6A right). Microvessel sprouting from P10 *Elk3(−/−)* aortae was also reduced compared to control animals (Figure 6C). This analysis demonstrates that Elk3 contributes to endothelial cell sprouting and migration.

## Discussion

In this study we show that *Elk3(−/−)* mice display a dual retinal phenotype: (i) transient impairment in post-natal development of the superficial vascular plexus, paralleled by (ii) a lasting retinal arterial tortuousity in animals 4 weeks of age and older. Arterial tortuousity is found in the retinae of adult Elk3 deficient mice with 100% penetrance. These phenotypic features are not displayed by knockout animals of the paralogues *Elk1* and *Elk4* (Table 3). This shows that Elk3 has distinct, non-redundant functions relative to Elk1 and Elk4 in retinal vascular development, as was similarly indicated for thymocyte development [23] and adhesion of cultured cells [35].

**Table 3:** Comparison of retinal phenotypes in the different knockout mouse models.

Knockout mouse model	*Srf ^iECKO^*	*Mrtf-a^(−/−)^ Mrtf-b ^iECKO^*	*Elk1/Elk4 ^dKO^*	*Elk3 KO*
	**Post-natal analysis**			
**Radial outgrowth**	drastically reduced^#^	drastically reduced^#^	unaffected^#^	transiently reduced at early stages^*^
**Tip cell morphology**	drastically altered^#^	drastically altered^#^	unaffected^#^	unaffected^*^
**Distal microaneurysms**	present^#^	present^#^	absent^#^	absent^*^
**Deep plexi**	absent^#^	absent^#^	present^#^	present^*^
	**Adult analysis**			
**Neovascular lesions**	present^#^	present^§^	absent^*^	absent^*^
**Tortuous arteries**	absent^#^	absent^§^	absent^*^	present^*^

(^*^ this study, ^#^ published in Weinl et al., 2013, ^§^ unpublished observations).

At the molecular level, we find *Elk3(−/−)* mice to display reduced retinal RNA expression of the angiogenic receptor Tie1, at P6, P10 and adult stage. The co-receptor Tie2 was found to have lower RNA levels at P6 but not P10 or adult retinae, while the cognate ligands Ang-1 and Ang-2 were expressed at normal RNA levels at all times investigated. These findings correlate with the arterial tortuousity phenotype shared by *Elk3(−/−)* and *Ang-2(−/−)* mice [30], indicating in both knockout models an impaired functionality of the Tie/Ang receptor/ligand signalling system. Of note, endothelial Tie1 depletion in conditional knockout mice leads to decreased sprouting angiogenesis in the post-natal retinal vasculature [36], similar to *Elk3(−/−)* mice. Interestingly, this conditional phenotype was only seen when *Tie1* deletion was induced directly after birth but not when the deletion was induced between P7 and P9, suggesting a short window for the requirement of Tie1 during initial post-natal retinal angiogenesis, while – at the same time - arguing for Tie1 not being important in subsequent vascular remodelling [36]. Thus, the transient post-natal requirement for Tie1 in retinal angiogenesis, as revealed by conditional Tie1 depletion [36], is fully congruent with the transient impairment in retinal primary plexus development observed here in *Elk3(−/−)* mice, which also displayed reduced Tie1 expression. We thus hypothesize that impairment in the functionality of the Tie1/Ang-2 signaling system accounts – in part - for both phenotypic irregularities of *Elk3(−/−)* mice, namely transient inhibition of retinal primary plexus formation and adult retinal arterial tortuousity. We do not exclude potential contributions of the Tie2 co-receptor. We further hypothesize that Elk3, but not Elk1 or Elk4, is involved in transcriptional regulation of *Tie1* expression. *Tie1* may represent a direct or indirect Elk3 target gene. We note that approximately 1.6 kb upstream of the murine *Tie1* promoter, there is a canonical SRF-binding CArG-box with the sequence CCTTAATTGG. This element, however, is not conserved at this position in the human genome. Future studies will investigate whether a functionally relevant ternary complex of SRF and Elk3 is formed with this CArG-box *in vivo* and wether the postulated complex shows preference for Elk3 over Elk1 or Elk4. In light of the findings by D'Amico et al. [36], our data suggest that Elk3 represents a potential target for tumor therapy.

Alternatively, or in addition, reduced retinal expression at P6 and P10 of the immediate-early and proliferation-associated genes *Egr-1, Egr-2* and c-*fos* might have contributed to the transient delay in retinal angiogenesis of *Elk3(−/−)* mice. However, our measurements (Ki67 marker quantitation for proliferating cells at P6, vessel width at P6, P8 and adult animals) did not reveal any differences in proliferation of retinal endothelial cells at any of the investigated time points. We have therefore no direct experimental evidence for Elk3 impairment affecting proliferation of retinal endothelial cells.

In a separate study, human ELK3 was argued to inhibit angiogenic functions of HUVEC cells *in vitro* [37]. While this finding appears to differ with our report, the specific conditions and cells studied by Heo and Cho (2014) may have evoked the inhibitory role of Elk3, and could have involved Elk3-dependent changes in Tie1 expression in combination with inhibitory Ang-2 signaling, as suggested by this study.

The *Elk3(−/−)* mice differ from other mouse and rat models for tortuousity. In the rat ROP model, tortuousity is prevalent at young age and then decreases [38]. In contrast, *Elk3(−/−)* retinal arteries are completely normal in young animals, tortuousity is first obvious at four weeks of age and persists into later ages (2–8 months). The rat model of type-2 diabetic retinopathy displays tortuous retinal vessels that almost completely lack pericytes, microaneurysms and higher vessel diameters, and irregular retinal layering [39]. In contrast, *Elk3(−/−)* knockout retinal vessels have a normal smooth muscle coverage. Mutations in the *Col4a1* gene encoding procollagen type IVα1 result in tortuous arteries in mice with the C57BL/6J background [31]. We did not detect any significant changes in collagenIV levels between control and *Elk3(−/−)* knockout retinae as judged by antibody staining on adult retinal flat-mounts and qRT-PCR using whole retinal adult tissue. Tortuous vessels are commonly seen after injection of TNFα in mice [40], in a rat model of Oxygen Induced Retinopathy (OIR) [38] and in patients with cyanotic congenital heart disease [41]. Tortuousity might increase perfusion of avascular regions of the retina by reducing blood flow. All of these conditions are associated with increased VEGF expression. In contrast, *Vegf* RNA levels are normal in adult *Elk3(−/−)* knockout retinae displaying tortuousity. Furthermore, retinal layering and layer thickness are normal in *Elk3(−/−)* knockout mice, in contrast to the TNFα-injection mouse model [40].

Vessel tortuousity is observed in a number of human pathologies, for example in an aggressive form of ROP that additionally displays rapid retinal detachment, leakage of blood vessels and severe dilations [19]. In *Elk3(−/−)* knockout mice leakage of affected blood vessels was not detected by *in vivo* SLO angiography, which is consistent with unchanged VEGF levels. In some other human patients, changes in thickness of vessels are correlated with occurrence of abnormally shaped vessels. In patients with aortic isthmic coarctation, retinal arteriolar, but not venular diameter, was reduced [42], [43]. In the Elk3 deficient arteries, proliferation was unchanged, as we did not detect any change in diameter of abnormally shaped adult arteries. Venular tortuousity has been observed in some human patients diagnosed with aortic isthmic coarctation [42], [43] and in a screen for abnormalities of ocular fundi in monkeys [44]. Interestingly, only arteries are affected in the *Elk3(−/−)* eyes, as in the rat model of OIR [38], in humans with ATS (arterial tortuousity syndrome) [45], and in the mouse TNFα injection model [40]. Abnormally shaped vessels are sometimes correlated with vision loss. ERGs are altered in ROP eyes [38], TNFα injected mice [40], and monocular vision loss during an exercise marathon run [46]. However, some patients with tortuous blood vessels and elevated VEGF levels have no ocular complaints [41]. As revealed by ERG measurements, vision was not impaired in adult *Elk3(−/−)* knockout mice. Blood pressure or hypertension could be expected to affect arterial shape. However, human patients with aortic isthmic coarctation after operative repair had retinal arteriolar tortuousity, but no hypertension, and blood pressure was normal [42], [43], suggesting that vessel tortuousity is not necessarily correlated with hypertension. In agreement, we did not detect any abnormalities in blood pressure of *Elk3(−/−)* knockout animals.

Interestingly, a recent clinical characterization of FEVR patients (familial exudative vitreoretinopathy), using wide-field fluorescein angiography, revealed hitherto unrecognized, frequent vessel tortuousity among these patients [20]. We therefore note that post-natal impairments in retinal plexus formation and vessel tortuousity are shared pathological features of some human FEVR patients and murine knockout models of the SRF transcriptional system. The latter is evidenced by endothelial depletion of SRF itself [8], endothelial depletion of the SRF cofactors MRTF-A and –B [8], and constitutive depletion of the SRF cofactor Elk3 (this study). It remains an intriguing, but testable possibility that mutations in Elk3 alleles might contribute to FEVR pathology.

Using the aortic ring assay [33], we tested if endothelial cells of an origin other than the retinal blood vessel system were affected by the absence of Elk3. This turned out to be the case. Aortic rings explanted from P10 or adult *Elk3(−/−)* animals showed impairments in microvessel sprouting and tube formation. While this defect mirrors the post-natal delay in retinal angiogenesis of *Elk3(−/−)* mice, it does not reflect the transient nature of this effect. The observed impairment in *Elk3(−/−)* aortic ring adherence functions, leading to the formation of retraction bulbs, might however reflect specific functions of Elk3 in regulating adhesion-mediated cell behaviours [35].

In conclusion, this study shows that Elk3 has a distinct role in determining retinal RNA levels of immediate-early genes and genes encoding the angiogenic receptors Tie1 and Tie2. Elk3 is involved in ensuring proper radial outgrowth and arterial length in the mouse retina, which is consistent with previous *in vivo* and *in vitro* studies [21], [34]. Interestingly, the new *Elk3(−/−)* mouse model described in our study has a highly penetrant phenotype of tortuous vessels with distinct features that could be used to study mechanisms of angiogenesis and vessel formation and could thus lead to a better understanding of human pathologies displaying tortuous vessels, including ROP and FEVR.

## Supporting Information

Figure S1 **The targeting vector was made as follows.** The 5′ (4.3 kb), 3′ (3 kb) and inter-loxP (2.37 kb) fragments were PCR amplified on 129sv genomic DNA and sequentially subcloned in an ICS proprietary vector containing the LoxP sites and a Neo cassette flanked by FRT sites (Figure S1). The linearized construct was electroporated in 129S2/SvPas mouse embryonic stem (ES) cells. After selection, targeted clones were identified by PCR using external primers and further confirmed by Southern blotting with 5′ external probe. Two positive ES clones were injected into C57BL/6J blastocysts, and derived male chimeras gave germline transmission. The excision of the neomycin-resistance cassette was performed *in vivo* by breeding the chimeras with a Flp deleter line (C57BL/6J genetic background). The Flp transgene was segregated by breeding the first germ line mice with a wild type C57BL/6J animal. Constitutive KO mice were generated by breeding floxed-allele heterozygotes with a Cre deleter line followed by segregation in a further breeding step. The initial targeting vector contained a deletion of 104 bp (indicated by a red triangle), which was inconsequential regarding generation of the KO locus. Subseqent to Cre activation, deletion of genomic sequences extends from −1720 to +650 relative the major transcription start site (TSS, +1), which corresponds to the first nucleotide of Elk3-001 ENSMUST00000008542 (mouse GRCm38, Ensembl). The location of primers used for genotyping is indicated by P1, P3 and P4. Use of primers P1 and P3 detect the WT allele (PCR product size 363 bp), use of primers P3 and P4 amplify sequences surrounding the region that is deleted in the KO, and therefore detect a shorter fragment in Elk3 KO mice (PCR product size 230 bp, the 2480 bp product is not amplified under the PCR conditions used). Features are not drawn to scale. Primer sequences: primer P1: 5′- GGTTCCTCCTAGAAATCTCCCCAAG-3′; primer P3: 5′-TTTGCACTCAGGGTGTCTCCTCC-3′; primer P4: 5′-CACAGTTCACCTGATGGCTCACTC-3′. PCR conditions: 1×: 94°C 3 min. 2×: 94°C 1 min., 62°C 1 min., 72°C 1 min. 30×: 94°C 30 sec., 62°C 30 sec., 72°C 30 sec. 1×: 72°C 3 min. and cooling to 4°C.
(PDF)

Figure S2 **Measurement of arterial length on adult** ***Elk3(+/+)*** **wildtype and** ***Elk3(−/−)*** **knockout retinal flat-mount preparations.** For quantitative analysis, tortuous length (red line) and idealized length (yellow line) are measured in μm in both wildtype and knockout retinal flat-mounts stained with ILB4, and subsequently a tortuousity factor as defined as the ratio red line/yellow line is calculated and used to characterize tortuousity: a value close to 1 states no tortuousity (as shown for wildtype arteries), whereas a value > 1 is a hallmark of vessel tortuousity (as shown for knockout arteries). Values for WT retinae are normalized to 1. The value for knockout arteries with 1.06 (knockout arteries are on average 6% longer than wildtype arteries) is significantly different compared to WT as tested by Student's t-test statistics (p<0.001 *** as stated in Table 2). The measurements were performed on n = 9 WT retinae and n = 9 knockout retinae from different animals including 36 WT arteries and 30 knockout arteries in total.
(PDF)

Figure S3 **Blood pressure measurements in female and male** ***Elk3(+/+)*** **control and** ***Elk3(−/−)*** **knockout mice (n = number of animals analysed).** The data shown are means +/− s.e.m., ns not significant.
(PDF)

Figure S4 **Retinal function is not impaired in adult** ***Elk3*** **-deficient mice.** Electroretinographic data of adult *Elk3*(+/+) (black) and *Elk3(−/−)* mice (red). (A) Representative scotopic reponses at -2 (top) and 1.5 (middle) log cd*s/m^2^, as well as a photopic response at 1.5 log cd*s/m^2^ (bottom) flash intensity. Scotopic (B) and photopic (C) b-wave amplitudes from *Elk3* control mice and *Elk3*-deficient mice are plotted as a function of the logarithm of the flash intensity. In the box-and whisker-plot, boxes indicate the 25% and 75% quantile range, whiskers indicate the 5% and 95% quantiles, and the asterisks indicate the median of the data.
(PDF)

Figure S5 **Analysis of astrocyte migration visualized by GFAP and ILB4 co-staining on** ***Elk3(+/+)*** **wildtype and** ***Elk3(−/−)*** **knockout retinal flat-mount preparations of different post-natal ages.** No difference was observed in astrocyte migration towards the retinal periphery between *Elk3(+/+)* and *Elk3(−/−)* mice at all ages analysed (P4, 6 and 8). Number of analysed retinae of (WT/KO) genotype: P4 (10/6 retinae), P6 (8/8 retinae), P8 (12/8 retinae). ILB4 co-staining (not shown in this figure) was used to evaluate radial outgrowth of retinal blood vessels. Scale bars 100 µm.
(PDF)

Figure S6 **P6 retinal flat-mounts of** ***Elk3(+/+)*** **and** ***Elk3(−/−)*** **mice were co-stained with ILB4 (green) and collagenIV (red).** Overlay of both images results in the merge image (yellow). To quantify for vessel regression, number of vessels exclusively stained for collagenIV (as highlighted by white arrows), but not for ILB4, were counted per vascularized area. No difference was observed between both genotypes (ns not significant). Scale bars ILB4, CollagenIV and merge images 100 µm, zoom 50 µm.
(PDF)

Figure S7 **(A) Western Blot analysis for P-Cofilin levels of two representative pairs of** ***Elk3(+/+)*** **control and** ***Elk3(−/−)*** **knockout adult whole retinal tissue, GAPDH was used as a loading control.** (B) Quantitative Western Blot analysis of five pairs of *Elk3(+/+)* control and *Elk3(−/−)* knockout adult whole retinal tissue. P-Cofilin protein levels were calculated in relation to GAPDH (n = 5 independent experiments), ns not significant.
(PDF)

Table S1 **Primer sequences for qRT-PCR of mouse tissue.** Shown are forward (fw) and reverse (rev) sequences for analysed target genes. Gapdh was used as a housekeeping gene for normalization in all experiments.
(PDF)

Table S2 **Measurement of proliferation by Ki67 and ILB4 co-staining of P6 and measurement of blood vessel width on ILB4 stained P6 and P8** ***Elk3(+/+)*** **wildtype and** ***Elk3(−/−)*** **knockout retinal flat-mount preparations.** Retinal flat-mounts of *Elk3(+/+)* WT and *Elk3(−/−)* KO P6 animals were co-stained with ILB4 and Ki67 and photographed at 20× magnification. On these images, Ki67 positive endothelial cells per vessel length were measured. Subsequently, Ki67 positive endothelial cells per 100 µm were calculated and all values of the WT were normalized to 100%. ns  =  not significant as tested by Student's t-test (p>0.05). For measurement of width, P6 and P8 ILB4-stained retinal flat-mounts were analysed. For width measurement, arteries and veins were analysed separately by outlining blood vessel Area (A) and blood vessel Length (L) and calculating mean width by Length/Area (L/A), followed by normalization of WT values to 100% (ns  =  not significant).
(PDF)
