# Supplementary figures and images for: Elk3 Deficiency Causes Transient Impairment in Post-Natal Retinal Vascular Development and Formation of Tortuous Arteries in Adult Murine Retinae
Source: PLoS One. 2014 Sep 9;9(9):e107048. doi: 10.1371/journal.pone.0107048 (PMC4159304; doi:10.1371/journal.pone.0107048)

Figure S1

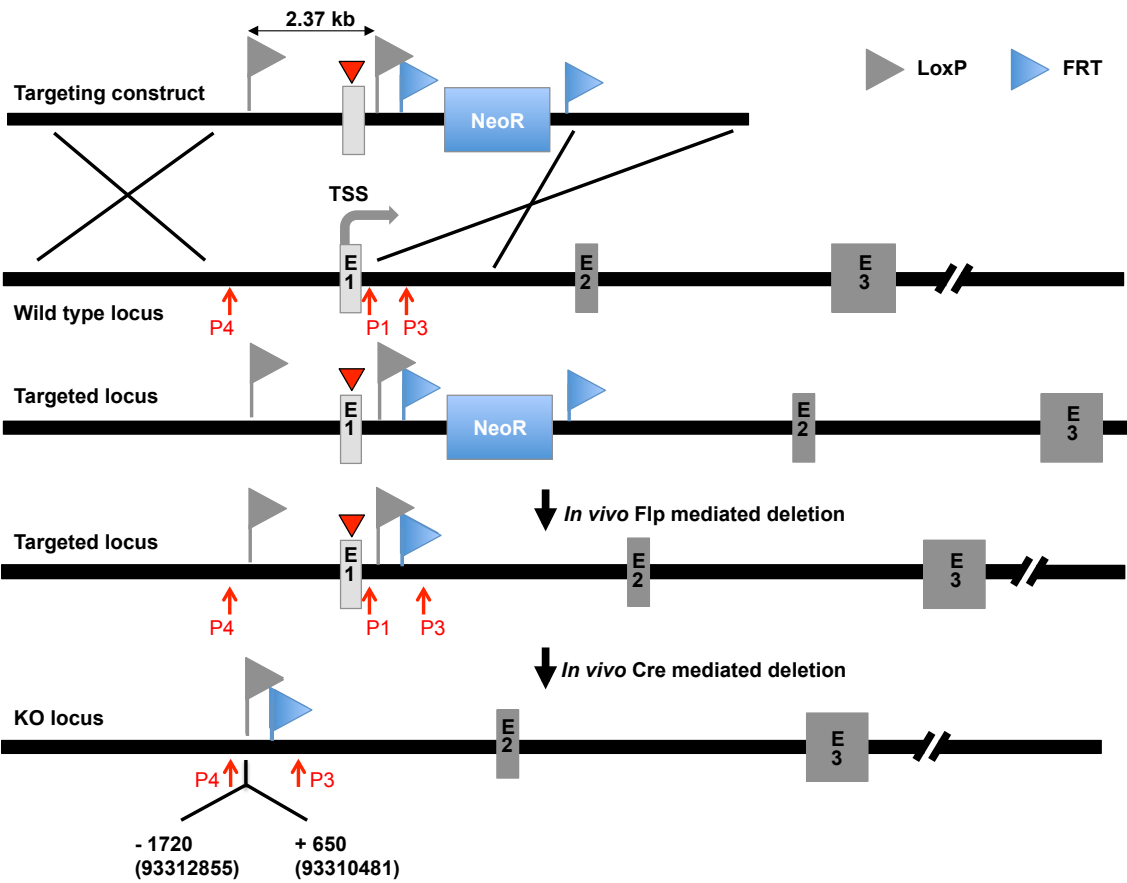

Supplement: Figure S1 — The targeting vector was made as follows. The 5′ (4.3 kb), 3′ (3 kb) and inter-loxP (2.37 kb) fragments were PCR amplified on 129sv genomic DNA and sequentially subcloned in an ICS proprietary vector containing the LoxP sites and a Neo cassette flanked by FRT sites (Figure S1). The linearized construct was electroporated in 129S2/SvPas mouse embryonic stem (ES) cells. After selection, targeted clones were identified by PCR using external primers and further confirmed by Southern blotting with 5′ external probe. Two positive ES clones were injected into C57BL/6J blastocysts, and derived male chimeras gave germline transmission. The excision of the neomycin-resistance cassette was performed in vivo by breeding the chimeras with a Flp deleter line (C57BL/6J genetic background). The Flp transgene was segregated by breeding the first germ line mice with a wild type C57BL/6J animal. Constitutive KO mice were generated by breeding floxed-allele heterozygotes with a Cre deleter line followed by segregation in a further breeding step. The initial targeting vector contained a deletion of 104 bp (indicated by a red triangle), which was inconsequential regarding generation of the KO locus. Subseqent to Cre activation, deletion of genomic sequences extends from −1720 to +650 relative the major transcription start site (TSS, +1), which corresponds to the first nucleotide of Elk3-001 ENSMUST00000008542 (mouse GRCm38, Ensembl). The location of primers used for genotyping is indicated by P1, P3 and P4. Use of primers P1 and P3 detect the WT allele (PCR product size 363 bp), use of primers P3 and P4 amplify sequences surrounding the region that is deleted in the KO, and therefore detect a shorter fragment in Elk3 KO mice (PCR product size 230 bp, the 2480 bp product is not amplified under the PCR conditions used). Features are not drawn to scale. Primer sequences: primer P1: 5′- GGTTCCTCCTAGAAATCTCCCCAAG-3′; primer P3: 5′-TTTGCACTCAGGGTGTCTCCTCC-3′; primer P4: 5′-CACAGTTCACCTGATGGCTC [file pone.0107048.s001.pdf]

Figure S2

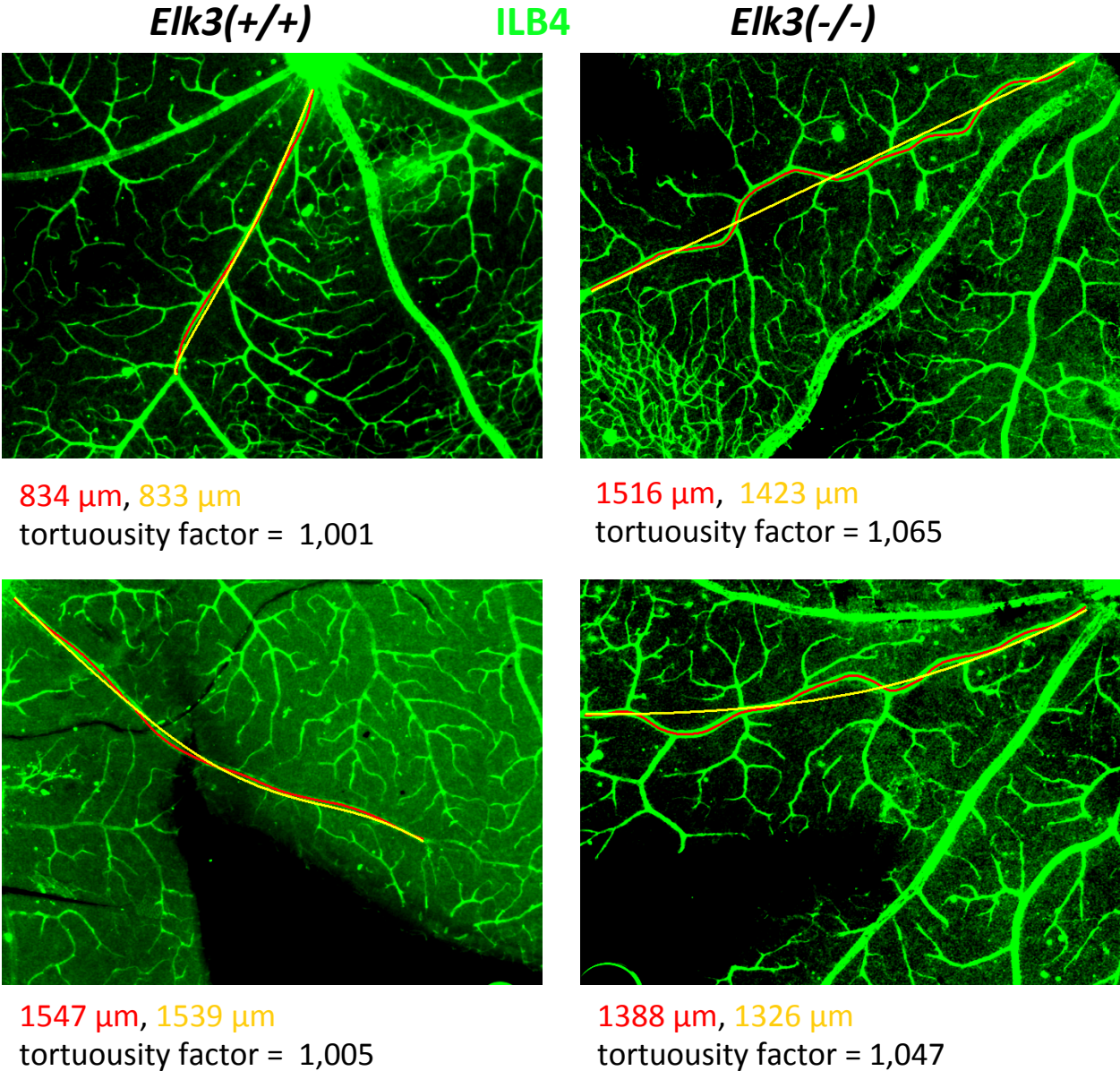

Supplement: Figure S2 — Measurement of arterial length on adult Elk3(+/+) wildtype and Elk3(−/−) knockout retinal flat-mount preparations. For quantitative analysis, tortuous length (red line) and idealized length (yellow line) are measured in μm in both wildtype and knockout retinal flat-mounts stained with ILB4, and subsequently a tortuousity factor as defined as the ratio red line/yellow line is calculated and used to characterize tortuousity: a value close to 1 states no tortuousity (as shown for wildtype arteries), whereas a value > 1 is a hallmark of vessel tortuousity (as shown for knockout arteries). Values for WT retinae are normalized to 1. The value for knockout arteries with 1.06 (knockout arteries are on average 6% longer than wildtype arteries) is significantly different compared to WT as tested by Student's t-test statistics (p<0.001 *** as stated in Table 2). The measurements were performed on n = 9 WT retinae and n = 9 knockout retinae from different animals including 36 WT arteries and 30 knockout arteries in total. (PDF) [file pone.0107048.s002.pdf]

Figure S3

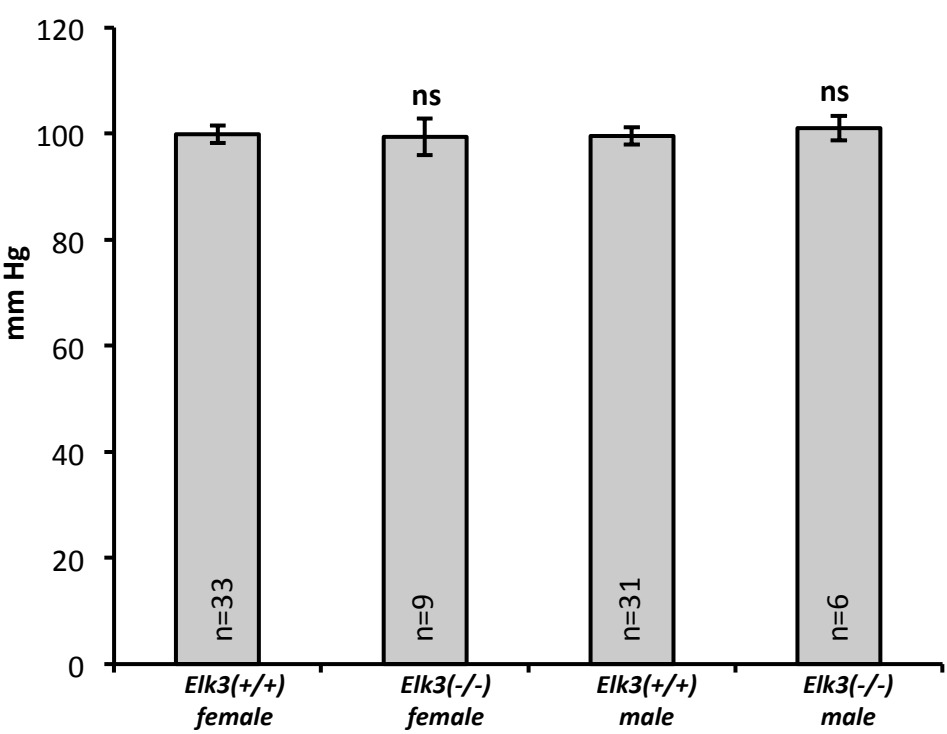

Supplement: Figure S3 — Blood pressure measurements in female and male Elk3(+/+) control and Elk3(−/−) knockout mice (n = number of animals analysed). The data shown are means +/− s.e.m., ns not significant. (PDF) [file pone.0107048.s003.pdf]

**Figure S4**

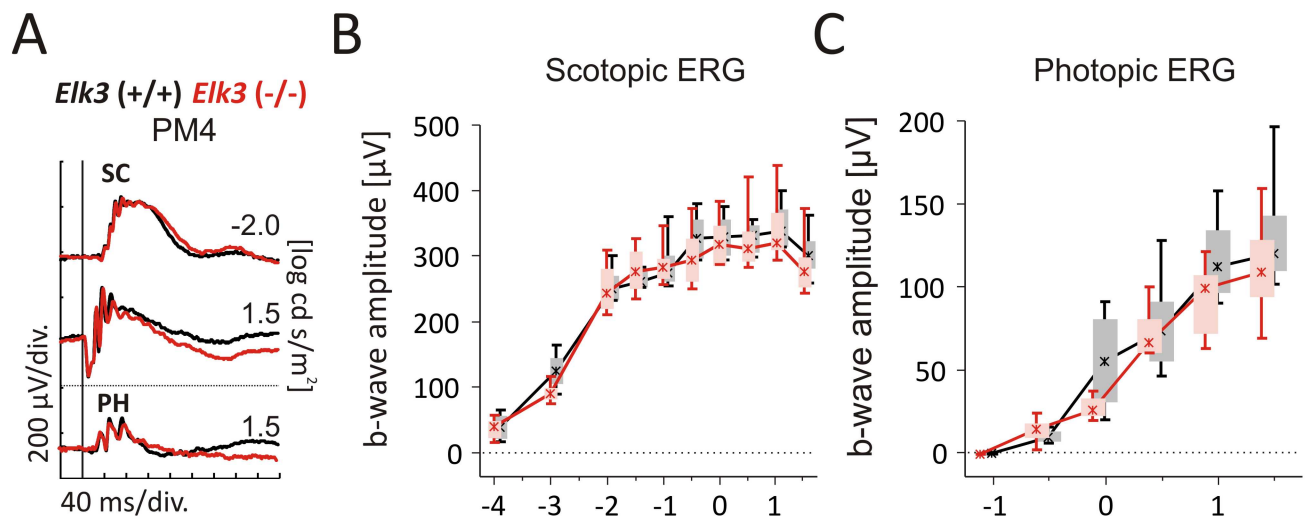

Supplement: Figure S4 — Retinal function is not impaired in adult Elk3 -deficient mice. Electroretinographic data of adult Elk3(+/+) (black) and Elk3(−/−) mice (red). (A) Representative scotopic reponses at -2 (top) and 1.5 (middle) log cd*s/m2, as well as a photopic response at 1.5 log cd*s/m2 (bottom) flash intensity. Scotopic (B) and photopic (C) b-wave amplitudes from Elk3 control mice and Elk3-deficient mice are plotted as a function of the logarithm of the flash intensity. In the box-and whisker-plot, boxes indicate the 25% and 75% quantile range, whiskers indicate the 5% and 95% quantiles, and the asterisks indicate the median of the data. (PDF) [file pone.0107048.s004.pdf]

Figure S5

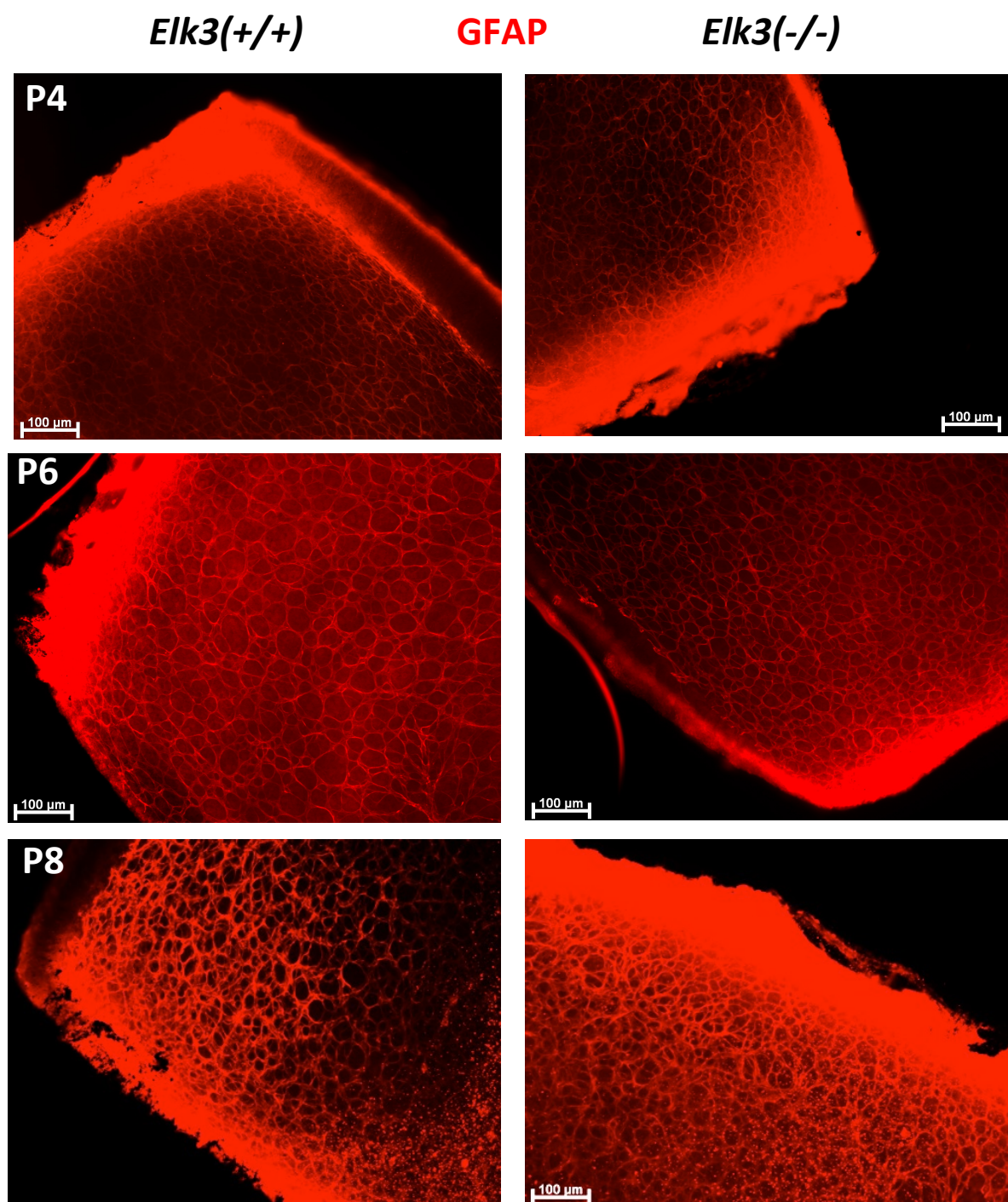

Supplement: Figure S5 — Analysis of astrocyte migration visualized by GFAP and ILB4 co-staining on Elk3(+/+) wildtype and Elk3(−/−) knockout retinal flat-mount preparations of different post-natal ages. No difference was observed in astrocyte migration towards the retinal periphery between Elk3(+/+) and Elk3(−/−) mice at all ages analysed (P4, 6 and 8). Number of analysed retinae of (WT/KO) genotype: P4 (10/6 retinae), P6 (8/8 retinae), P8 (12/8 retinae). ILB4 co-staining (not shown in this figure) was used to evaluate radial outgrowth of retinal blood vessels. Scale bars 100 µm. (PDF) [file pone.0107048.s005.pdf]

Figure S6

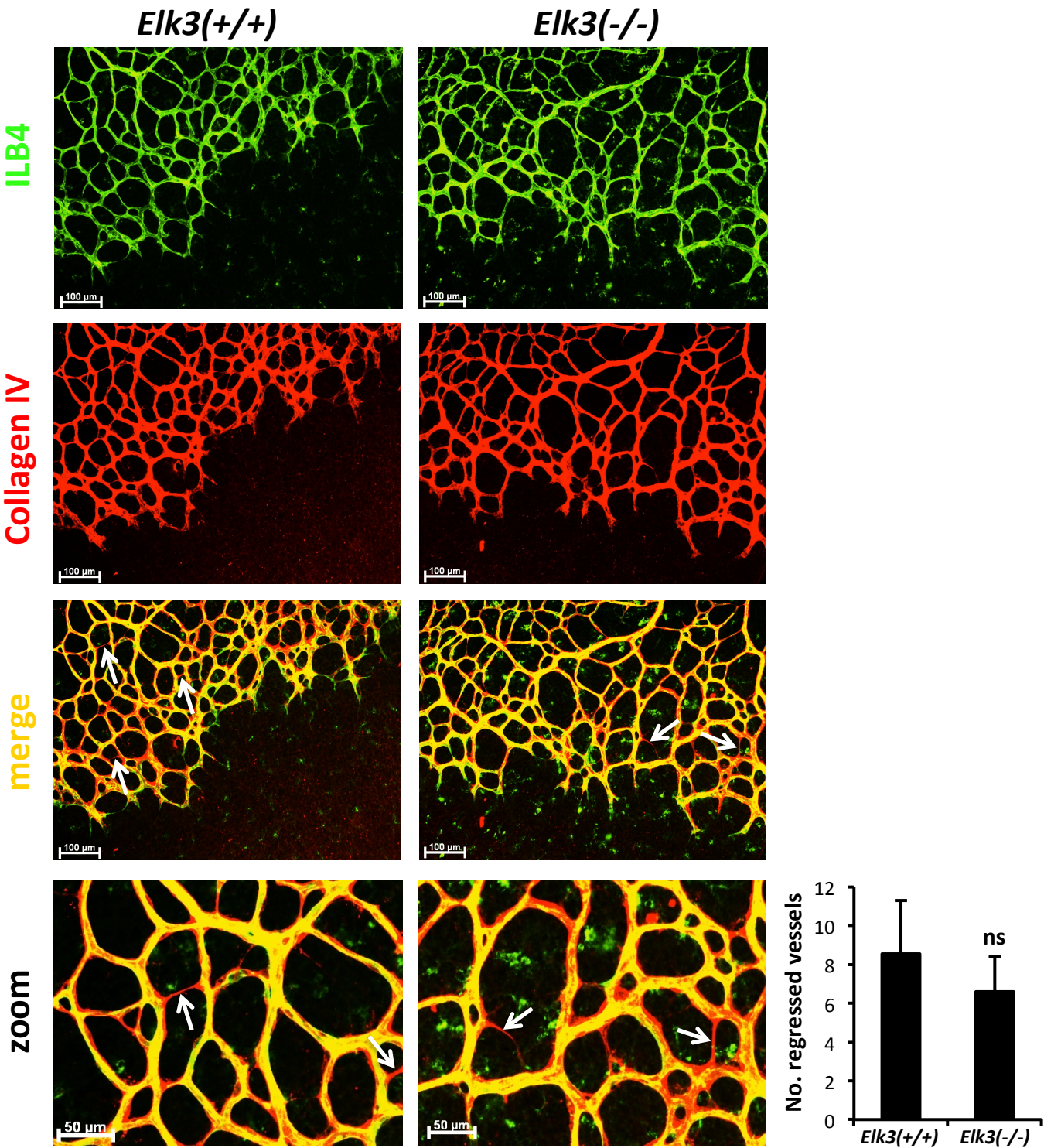

Supplement: Figure S6 — P6 retinal flat-mounts of Elk3(+/+) and Elk3(−/−) mice were co-stained with ILB4 (green) and collagenIV (red). Overlay of both images results in the merge image (yellow). To quantify for vessel regression, number of vessels exclusively stained for collagenIV (as highlighted by white arrows), but not for ILB4, were counted per vascularized area. No difference was observed between both genotypes (ns not significant). Scale bars ILB4, CollagenIV and merge images 100 µm, zoom 50 µm. (PDF) [file pone.0107048.s006.pdf]

# Figure S7

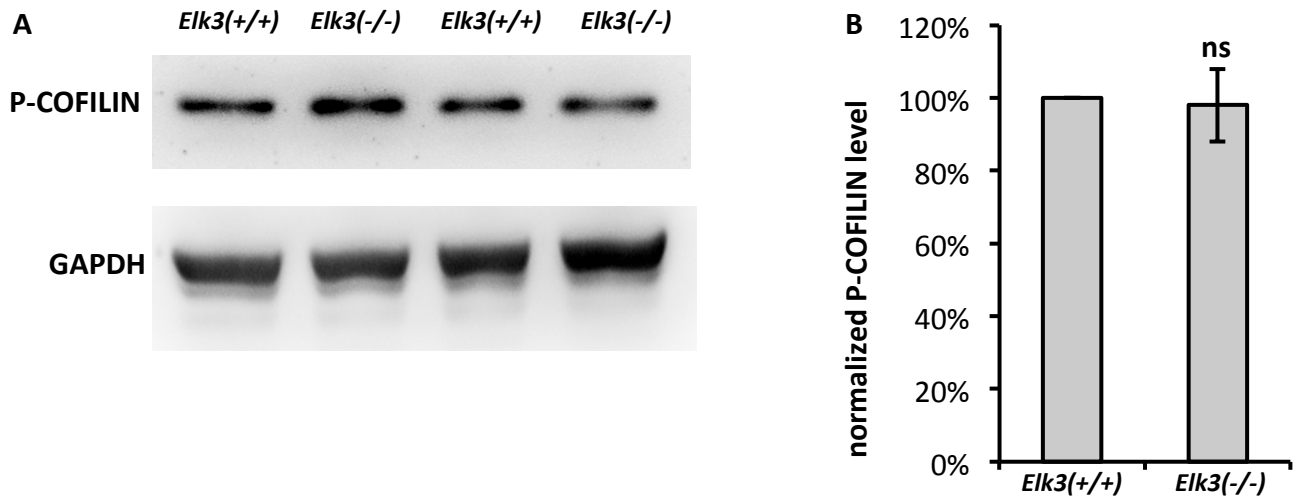

Supplement: Figure S7 — (A) Western Blot analysis for P-Cofilin levels of two representative pairs of Elk3(+/+) control and Elk3(−/−) knockout adult whole retinal tissue, GAPDH was used as a loading control. (B) Quantitative Western Blot analysis of five pairs of Elk3(+/+) control and Elk3(−/−) knockout adult whole retinal tissue. P-Cofilin protein levels were calculated in relation to GAPDH (n = 5 independent experiments), ns not significant. (PDF) [file pone.0107048.s007.pdf]
