# Supplementary material for: Elk3 Deficiency Causes Transient Impairment in Post-Natal Retinal Vascular Development and Formation of Tortuous Arteries in Adult Murine Retinae
Source: PLoS One. 2014 Sep 9;9(9):e107048. doi: 10.1371/journal.pone.0107048 (PMC4159304; doi:10.1371/journal.pone.0107048)
Supplement: Table S1 — Primer sequences for qRT-PCR of mouse tissue. Shown are forward (fw) and reverse (rev) sequences for analysed target genes. Gapdh was used as a housekeeping gene for normalization in all experiments. (PDF) [file pone.0107048.s008.pdf]

Table S1: Primer sequences for qRT-PCR

Primer Sequences for qRT-PCR	
Gapdh fw: TGG ATC TGA CGT GCC GC	Gapdh rev: TGC CGT CTT CAC CAC CTT C
Srf fw: CAC GAC CTT CAG CAA GAG GAA	Srf rev: CAA AGC CAG TGG CAC TCA TTC
β-actin fw: AGA GAG GTA TCC TGA CCC TGA AGT	β-actin rev: CAC GCA GCT CAT TGT AGA AGG TGT
c-fos fw CTT GCC CCT TCT CAA CGA	c-fos rev: GCT CCA CGT TGC TGA TGC T
Vegf-r2 fw: GAT GCC CGA CTC CCT TTG A	Vegf-r2 rev: CGA AAG ACC ACA CAT CGC TCT
Vegf-r1 fw: AGC CTA CCT CAC CGT GCA AG	Vegf-r1 rev: AAA AGA GGG TCG CAG CCA C
Vegf-a fw: TCA CCA AAG CCA GCA CAT AG	Vegf-a rev: TTG ACC CTT TCC CTT TCC TC
Elk3 fw: CTCAGGATCCCTCACTCCAG	Elk3 rev: GGCATGTGACCGTTGAGCAG
Egr-1 fw: GCC GAG CGA ACA ACC CTA T	Egr-1 rev: TCC ACC ATC GCC TTC TCA TT
Egr-2 fw: GTT GAC TGT CAC TCC AAG AAA TGG	Egr-2 rev: AGC GCA GCC CTG TAG GC
CollagenIV fw: CGGAGGAAGAAACTGCTCTG	CollagenIV rev: CACCAGTTGGACCCTTGTCT
Elk1 fw: TGCTCCCCACACATACCTTGA	Elk1 rev: ACTGGACGGAAACTGGAAGGA
Elk4 fw: GTGACAACGCCTGCCAAAA	Elk4 rev: GCCAGAATGGATATGGAGGCT
Ang-1 fw: AGCTACCAACAACAACAGCA	Ang-1 rev: GCAAAGGCTGATAAGGTTATGA
Ang-2 fw: AGCCACGGTCAACAACTCGC	Ang-2 rev: TTCTTCTTTACGGATAGCAAC
Tie-1 fw: CCCCACTGGTCTCCTTTAG	Tie-1 rev: AATGGCAGACCAGGCAATC
Tie-2 fw: GAAGTCGAGAGGCGATCCC	Tie-2 rev: GTTGACTCTAGCTCGGACTGT
