# Supplementary material for: Elk3 Deficiency Causes Transient Impairment in Post-Natal Retinal Vascular Development and Formation of Tortuous Arteries in Adult Murine Retinae
Source: PLoS One. 2014 Sep 9;9(9):e107048. doi: 10.1371/journal.pone.0107048 (PMC4159304; doi:10.1371/journal.pone.0107048)
Supplement: Table S2 — Measurement of proliferation by Ki67 and ILB4 co-staining of P6 and measurement of blood vessel width on ILB4 stained P6 and P8 Elk3(+/+) wildtype and Elk3(−/−) knockout retinal flat-mount preparations. Retinal flat-mounts of Elk3(+/+) WT and Elk3(−/−) KO P6 animals were co-stained with ILB4 and Ki67 and photographed at 20× magnification. On these images, Ki67 positive endothelial cells per vessel length were measured. Subsequently, Ki67 positive endothelial cells per 100 µm were calculated and all values of the WT were normalized to 100%. ns = not significant as tested by Student's t-test (p>0.05). For measurement of width, P6 and P8 ILB4-stained retinal flat-mounts were analysed. For width measurement, arteries and veins were analysed separately by outlining blood vessel Area (A) and blood vessel Length (L) and calculating mean width by Length/Area (L/A), followed by normalization of WT values to 100% (ns = not significant). (PDF) [file pone.0107048.s009.pdf]

Table S2: Measurement of proliferation via Ki67 and ILB4 co-staining on P6 and measurement of blood vessel width on ILB4 stained P6 and P8 P6 *Elk3(+/+)* wildtype and *Elk3(-/-)* knockout retinal flat-mount preparations

		**Ki67 positive endothelial cells / 100 μm**	
		Elk3(+/+) (n=9 retinae)	***Elk3(-/-)*** (n=10 retinae)
**P6 arteries**		100% (42 arteries)	98.75% (± 9.8%) p=0.91 ns (45 arteries)
**P6 veins**		100% (39 veins)	92% (± 4.7%) p=0.19 ns (45 veins)
		**Measurement of blood vessel width**	
**P6** (19 WT retinae) (17 KO retinae)	**arteries**	100% (96 arteries)	106% (±3%) p=0.14 ns (84 arteries)
	**veins**	100% (81 veins)	96.7% (±4%) p=0.45 ns (87 veins)
**P8** (8 WT retinae) (10 KO retinae)	**arteries**	100% (33 arteries)	101% (±5%) p=0.77 ns (38 arteries)
	**veins**	100% (25 veins)	95% (±3%) p=0.08 ns (38 veins)
